# Management of Venous Thromboembolism for Patients With Hematologic Malignancies

**Published:** 2017-04-01

**Authors:** Rowena N. Schwartz

**Affiliations:** James L. Winkle College of Pharmacy, University of Cincinnati, Ohio

## Abstract

Advanced practitioners are an integral part of the cancer care team. Therefore, it is imperative they are knowledgeable of risk factors associated with venous thromboembolism in the oncology setting, including signs or symptoms and management.

Coagulation in cancer is a fascinating topic. Venous thromboembolism hits upon all three of the main issues we talk about in oncology: treatment of the disease, complications of the disease, and comorbidities," said Rowena N. Schwartz, PharmD, BCOP, of the University of Cincinnati. At JADPRO Live 2016, Dr. Schwartz described the landscape of venous thromboembolism (VTE) in patients with hematologic malignancies, which includes deep vein thrombosis (DVT) and pulmonary embolism (PE).

Hemostasis is the complex process of maintaining the integrity of the circulatory system following damage to blood vessels. Hemostatic clots are those localized to the vessel wall. Thrombotic clots impair the blood flow.

## Etiology of Thrombosis in Cancer

The etiology of thrombosis in cancer is based on the three main factors of "Virchow’s triad": circulatory stasis, endothelial injury, and the hypercoagulable state. The vessel wall is injured, vasoconstriction occurs, platelets aggregate upon the release of natural activators of hemostasis, and the release of other factors causes the formation and adhesion of clots. From the clot comes the cascade of coagulation, with its thrombin formation, fibrin formation, and then stabilization of the clot.

The natural activators and inhibitors of hemostasis impact hemostasis at various points along the coagulation cascade. Activators include von Willebrand factor; collagen; tissue factor; tissue plasminogen activator; and factors VIIa, VIIIa, IXa, Va, Xa, and XIIIa. Inhibitors include antithrombin, heparin, thrombomodulin, protein C, protein S, tissue factor pathway inhibitor, and plasminogen activator inhibitor-1.

## Risk of VTE in Patients With Cancer

Not all malignancies are equal in terms of VTE risk. "The pathophysiology of increased risk of clots in patients with cancer is determined somewhat by the cancer itself," Dr. Schwartz revealed.

Relative risks appear highest for uterine cancer, brain tumors, leukemia, and pancreatic cancer and to a lesser degree lymphoma, stomach cancer, and colon cancer. It is important to note that the risk in some malignancies is increased only in certain subtypes.

"In practice, you will identify the patients most at risk in your population," she said. "I encourage you to look for the risks in your own patients."

In the general population, risk is associated with increased age, history of VTE, vascular stasis, hypercoagulable state, and certain medications. In patients with cancer, risks can be patient-related (potentially modifiable), cancer-related, and treatment-related (Table 1).

**Table 1:**
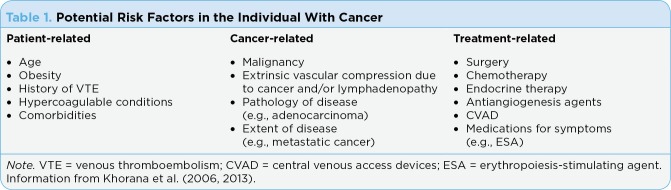
Potential Risk Factors in the Individual With Cancer

To help identify patients at risk, the Khorana score (Table 2) is a simple model based on a collection of baseline clinical and laboratory variables—type of cancer, body mass index (BMI), and complete blood cell count (platelet, leukocyte, hemoglobin; Khorana, Kuderer, Culakova, Lyman, & Francis, 2008). Patients who score ≥ 3 have a high risk, which falls somewhere between 7% and 41%.

**Table 2:**
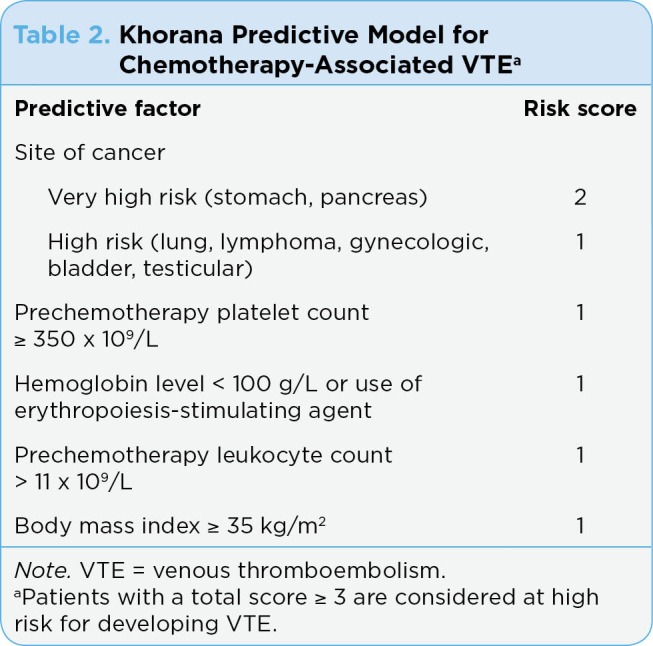
Khorana Predictive Model for Chemotherapy-Associated VTE^a^

## Recognition of VTE

Recognition of DVT can be complicated by the fact that its symptoms may overlap with those of cancer and certain medications (nonsteroid anti-inflammatory agents, antiemetics) and also may mask symptoms. Patients may have swelling of facial areas or extremities; pain, warmth, and heaviness in extremities; unexplained calf cramping; and catheter dysfunction.

"Be aware of the signs, identify risk factors for certain populations, and don’t just evaluate patients once," suggested Dr. Schwartz. "Since we are now seeing cancer patients over years, down the line they may need to be evaluated again for VTE risk."

Diagnostic studies include duplex venous ultrasonography, contrast-enhanced computed tomography (CT), magnetic resonance imaging (MRI), standard venography, and serum D-dimer.

The signs and symptoms of PE can also be challenging to evaluate. Symptoms may include cough, back pain, chest pain or tightness, shortness of breath, dyspnea on exertion, palpitations, hemoptysis, dizziness, and syncope. Clinical signs are tachypnea, tachycardia, diaphoresis, distention of neck veins, cyanosis, hypotension, and radiographic evidence.

"Recognition of PE is especially problematic in outpatients. You must educate patients and their families to recognize these symptoms and call you," she said.

Modalities used for evaluation include CT angiography, ventilation/perfusion lung scan, and, when thrombolytic extraction or therapy is anticipated, pulmonary angiography.

A predictive model can help determine a person’s chemotherapy-associated risk of VTE. The Khorana model considers the site of the primary cancer, prechemotherapy platelet and leukocyte counts, hemoglobin level, use of erythropoiesis-stimulating agent, and BMI (Table 2).

In addition, D-dimer is often elevated in cancer and therefore may not be discriminatory. Similarly, probability assessment using the Wells model (a score of 3+ indicates high risk) has questionable validity in patients with cancer (Wells et al., 1997, 2000). There may be some role for clinical decision-support tools, such as the Pulmonary Embolism Rule-Out Criteria (PERC).

However, Dr. Schwartz cautioned against relying on predictive models, which, aside from the Khorana score, have not been validated in the cancer population.

## Management Options for VTE

The therapeutic aim is to manipulate factors associated with the anticoagulation cascade, primarily using one of the pharmacotherapy options (Table 3).

**Table 3:**
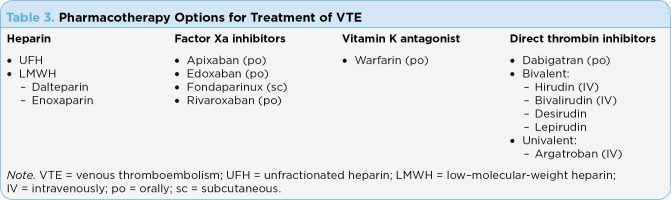
Pharmacotherapy Options for Treatment of VTE

Heparin is a naturally occurring polysaccharide that inhibits coagulation—the process that leads to thrombosis. Natural heparin consists of molecular chains of varying lengths (molecular weights). Heparin includes unfractionated heparin (UFH) and low–molecular-weight heparin (LMWH), which is about one-third the size of UFH. Oral anticoagulants include the novel and new oral anticoagulants, the direct oral anticoagulants, and a target-specific oral anticoagulant.

Heparin works by binding antithrombin and thrombin, producing an effect on thrombin and factor Xa. Unfractionated heparin is a small molecule; it does not bind thrombin but binds antithrombin-3. Low–molecular-weight heparin is a smaller molecular and has most of its effect on factor Xa, with less of an effect on thrombin.

"The key thing with heparin is its fast onset. It starts working almost immediately, and because of its short half-life, when you stop giving it, its action stops. This is a good option for the patient who needs anticoagulation for a procedure," indicated Dr. Schwartz.

Adverse effects include the potential for bleeding, osteoporosis (with long-term use), skin reactions, and heparin-induced thrombocytopenia (HIT). Heparin-induced thrombocytopenia, which can occur in up to 5% of patients, is characterized by a reduction in platelet count > 50% from baseline prior to heparin, hypercoagulability, and heparin-dependent platelet-activating immunoglobulin antibodies. Heparin-induced thrombocytopenia can be delayed (after cessation of heparin) or autoimmune (in the absence of heparin).

"Cancer patients have received so much heparin, they can develop HIT almost immediately," she noted.

Heparin-induced thrombocytopenia is treated by discontinuation of all heparin and initiation of alternative anticoagulation (although warfarin should be avoided with acute HIT).

For bleeding associated with UFH, discontinue heparin, transfuse, give supportive care, and, depending on when and how much heparin was given, reverse the anticoagulant effect with protamine sulfate. Unfractionated heparin levels are monitored by antifactor Xa activity and activated partial thromboplastin time.

Low–molecular-weight heparin has less inhibitory activity against thrombin (factor IIa) than does UFH. Its principal effect is on factor Xa and to a lesser degree thrombin. It is a smaller and more predictable molecule, whose antifactor Xa activity peaks 3 to 4 hours after a subcutaneous dose. Since it has renal elimination, LMWH is not appropriate for patients with renal dysfunction. The incidence of HIT is less (< 1%) than with UFH.

The two LMWH products, both given subcutaneously, are dalteparin (Fragmin) and enoxaparin. With dalteparin, the dose for thromboprophylaxis is 5,000 U/day, and the dose for treatment is 200 U/kg. For enoxaparin, the doses are 40 mg daily and 1 mg/kg daily, respectively. For monitoring, factor Xa activity can be used in select situations, but it is not an optimal means.

"The main problem with LMWH is that patients don’t want to inject themselves," Dr. Schwartz indicated. "So, it might be best for the patient to have an anticoagulant that is maybe not as effective, but one the patient will actually take."

## FACTOR Xa INHIBITORS

Factor Xa inhibitors—apixaban (Eliquis), edoxaban (Savaysa), rivaroxaban (Xarelto), fondaparinux—have had limited use in oncology. Only fondaparinux has evidence of efficacy as prophylaxis and treatment of DVT and PE in patients with cancer (National Institutes of Health, 2016). It is given once a day, subcutaneously, with doses based on weight. Fondaparinux is associated with a very low risk of HIT, making it a good option for patients with this risk for HIT.

Rivaroxaban, apixaban, and edoxaban are direct oral anticoagulants, which selectively and potently inhibit factor Xa. Their consistent efficacy has led to their increasing use in many clinical situations, except oncology. Bioavailability, peak effects, half-life, and various other factors differ among these three agents, giving them diverse advantages and disadvantages and uses. Their main advantages are their specificity, lack of required blood monitoring, lack of cross-reactivity with HIT antibodies, minimal drug interactions (at least, less than with warfarin), and relatively long half-life.

Direct thrombin inhibitors include dabigatran (Pradaxa), which is orally administered; lepirudin, argatroban, and bivalirudin, which are given intravenously; and desirudin (Iprivask), which is subcutaneously delivered. The bioavailability of dabigatran can be increased by chilling or crushing the pellets, and it is renally eliminated.

Dabigatran itself is not an anticoagulant but is converted to its active agent. It is poorly bioavailable (6.5%), but if chilled or crushed, its bioavailability increases (75%), producing an anticoagulation effect. It is renally eliminated; therefore, it is difficult to use in patients with renal insufficiency.

## WARFARIN: THE ’OLD’ ORAL ANTICOAGULANT

As a vitamin K antagonist, warfarin blocks the recycling of vitamin K, which prevents the production of clotting factors and propagation of the clot. Warfarin also blocks regulatory anticoagulant proteins (proteins C, S, Z).

According to a MarketScan Research Database analysis of newly diagnosed cancer patients, warfarin remains the most utilized anticoagulant for the outpatient treatment of VTE (Khorana et al., 2016). "It’s mainly about cost and the fact that patients don’t like to stick themselves," she said.

Initially, warfarin should be given concomitantly with UFH, LMWH, or fondaparinux for at least 5 days, and an international normalized ratio (INR) ≥ 2 should be achieved. "Always get a baseline INR. If you assume you know the patient’s ability to clot by looking at him, you can overdose him," stated Dr. Schwartz. "Also, remember that responses to warfarin can fluctuate. Anything that can change the dose (such as new medications) should be communicated to you by the patient or family."

The INR target range is determined by patient factors and indications. A common INR target range for VTE is 2 to 3.

Factors that may affect the INR include diarrhea, nausea, vomiting, diet, alcohol consumption, thyroid and liver function, medications, nonadherence, and activity level. When combining warfarin with drugs that may increase bleeding (nonsteroidal anti-inflammatory agents, aspirin, clopidogrel, chemotherapy), caution is advised. Since warfarin targets vitamin K–dependent clotting factors, the amount of vitamin K in the diet can also impact its effect.

## NONPHARMACOLOGIC OPTIONS

Placement of an inferior vena cava filter to prevent DVT from traveling to the lungs is another management strategy—but one with potential complications. Retrievable filters are strongly preferred and can be considered when therapeutic anticoagulation is absolutely contraindicated; when anticoagulation fails; and in patients who are nonadherent to prescribed anticoagulation, in whom PE would be life-threatening, and who have had multiple PEs and chronic pulmonary hypertension.

Thrombolytic agents are also "in vogue again," according to Dr. Schwartz, now that they are catheter-directed, and may be beneficial in subsets of patients.

## MANAGEMENT CONSIDERATIONS

There are absolute and relative contraindications to anticoagulation therapy in patients with hematologic cancer, "but [it is important to] understand that a patient who cannot get anticoagulation at one point may be able to, with other changes," explained Dr. Schwartz.

Absolute contraindications include recent central nervous system bleeding, an intracranial or spinal lesion with a high risk for bleeding, and active major bleeding. Relative contraindications include chronic, clinically significant measurable bleeding for more than 48 hours, thrombocytopenia, severe platelet dysfunction, recent major surgery carrying a high risk for bleeding, underlying hemorrhagic coagulopathy, a high risk for falls (head trauma), neural anesthesia/lumbar puncture, and interventional spine and pain procedures.

Prophylaxis for VTE should be considered for at-risk populations, including hospitalized patients (adult medical and surgical patients, cancer patients), ambulatory cancer patients recovering from high-risk abdominal or pelvic cancer surgery, and patients with multiple myeloma receiving an immunomodulatory drug.

Patients with cancer who develop VTEs should be immediately treated for at least 5 to 7 days with LMWH, UFH, or fondaparinux, after which they can be considered for chronic therapy with LMWH or warfarin (target INR, 2–3). The direct oral anticoagulants and direct thrombin inhibitors are not yet recommended in this setting, although their use is increasing.

The usual treatment duration is 3 to 6 months, but if the cancer recurs, anticoagulation should be restarted. For catheter-associated thrombosis, anticoagulation should be continued while the catheter is in place.

"The duration of active cancer is the duration of treatment, and this is a challenging discussion with patients," Dr. Schwartz noted. "Providers should continue to discuss the risks and benefits of anticoagulation, to determine the appropriate duration of therapy."

In patients who develop VTEs despite therapeutic anticoagulation, providers should evaluate whether their current anticoagulation is optimal (agent, dose, regimen, adherence) and whether new clinical factors have emerged; then they should correct any deficiencies or factors they identify. The INR could be increased with warfarin, the dose of LMWH can be increased, or the agent can be switched.

When reversal is required, providers should consult the National Comprehensive Cancer Network Guidelines and should already be familiar with the availability of the products and their mechanisms of action. Withholding of active treatment can be considered when there is no therapeutic advantage, no palliative benefit, an unreasonable burden of treatment, and patient refusal.
